# Aptameric Fluorescent Biosensors for Liver Cancer Diagnosis

**DOI:** 10.3390/bios13060617

**Published:** 2023-06-04

**Authors:** Seonga Park, Euni Cho, Sy-Tsong Dean Chueng, June-Sun Yoon, Taek Lee, Jin-Ho Lee

**Affiliations:** 1 School of Biomedical Convergence Engineering, Pusan National University, Yangsan 50612, Republic of Korea; 2 Department of Information Convergence Engineering, Pusan National University, Yangsan 50612, Republic of Korea; 3 Vitale Biotechnology, Inc., 700 West Park Ave, Perkasie, PA 18944, USA; deanchueng@gmail.com; 4 Department of Agricultural Convergence Technology, Jeonbuk National University, Jeonju 54896, Republic of Korea; 5 Department of Chemical Engineering, Kwangwoon University, Seoul 01897, Republic of Korea; 6 Department of Physiology, School of Medicine, Pusan National University, Yangsan 50612, Republic of Korea

**Keywords:** aptamer, biosensors, liver cancer, Förster resonance energy transfer, metal-enhanced fluorescent

## Abstract

Liver cancer is a prevalent global health concern with a poor 5-year survival rate upon diagnosis. Current diagnostic techniques using the combination of ultrasound, CT scans, MRI, and biopsy have the limitation of detecting detectable liver cancer when the tumor has already progressed to a certain size, often leading to late-stage diagnoses and grim clinical treatment outcomes. To this end, there has been tremendous interest in developing highly sensitive and selective biosensors to analyze related cancer biomarkers in the early stage diagnosis and prescribe appropriate treatment options. Among the various approaches, aptamers are an ideal recognition element as they can specifically bind to target molecules with high affinity. Furthermore, using aptamers, in conjunction with fluorescent moieties, enables the development of highly sensitive biosensors by taking full advantage of structural and functional flexibility. This review will provide a summary and detailed discussion on recent aptamer-based fluorescence biosensors for liver cancer diagnosis. Specifically, the review focuses on two promising detection strategies: (i) Förster resonance energy transfer (FRET) and (ii) metal-enhanced fluorescence for detecting and characterizing protein and miRNA cancer biomarkers.

## 1. Introduction

Cancer has emerged as a major life-threatening disease worldwide in the last few decades [1]. Although breast, lung, and prostate cancers have a higher incidence rate, lung, liver, and stomach cancers claim the most lives per year [2]. Liver cancer is particularly concerning due to the high mortality rate compared to the incidence rate [3]. While periodic check-ups are necessary to prevent cancer risk factors and monitor the prognosis and recurrence, liver cancer is often diagnosed at late stages when the chance of cure and survival is relatively low [4]. The poor prognosis is because liver cancer has an insidious onset and lacks specific early markers leading to difficulties in early diagnosis, rapid progression, and lack of targeted therapies, which lead to poor patient survival [5]. Thus, to address these concerns, research has been aimed at developing a more sensitive and accurate detection technique for the early diagnosis and successful treatment of cancer [6].

Various approaches, including electrical/electrochemical, optical, and mechanical detection strategies, have demonstrated great promise in cancer diagnosis [7,8,9,10]. Optical biosensing platforms have garnered widespread attention as a preferred technology for monitoring cancer-related biomolecules and biological processes due to their portability, rapidity, and cost-effectiveness, making them an attractive choice in cancer research [11,12,13]. In particular, fluorescent-based biosensing platforms are crucial in cancer diagnosis owing to their high sensitivity, selectivity, rapid responsivity, and simplicity [14,15]. Representative fluorescence-based biosensors utilize phenomena including Förster resonance energy transfer (FRET) and metal-enhanced fluorescence (MEF). FRET is a nonradiative energy transfer phenomenon generated through dipole–dipole coupling between two fluorophores [16]. MEF is a method that enhances the FRET of a fluorophore due to its interactions with metallic nanostructure surfaces, allowing for highly sensitive biomarker detection [17,18].

On the other hand, cancer development involves several biological changes at a molecular level, including mutation and abnormal expressions of DNA, RNA, protein, etc., resulting in malignancy. Thus, the precise quantitation of these changes at the molecular level is considered an essential parameter for diagnosis and prognosis [19]. However, due to the varied features of cancer biomarkers, such as expression level and binding moiety based on their structural difference, a receptor, a recognition element of the target molecule, is critical for accurate and sensitive cancer biomarker detection. In this regard, an aptamer that can recognize a variety of targets, including oligonucleotides, proteins, small molecules, and even exosomes and whole cells, has emerged and has been extensively utilized to develop biosensors for early monitoring of various cancer biomarkers [14].

Composed of short single-stranded DNA or RNA molecules and designed through the SELEX process [20], aptamers can specifically recognize a biological target with high selective affinity through weak interaction, including hydrogen bonding, van der Waals forces, electrostatic interactions, and shape complementarities to act similar to antigen–antibody interactions [20,21]. Moreover, aptamers can be easily synthesized for various biological targets and functionalized with chemical functional groups such as fluorophores, making them excellent target recognition molecules for developing biosensors [8,14,22].

**Figure 1:**
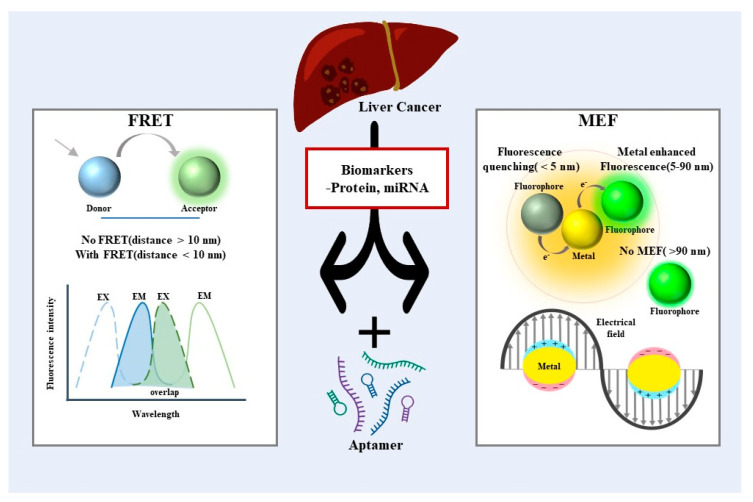
Schematic illustration of aptameric fluorescent biosensors for liver cancer diagnosis.

While many reviews address cancer diagnosis platforms, the development of biosensors for precise liver cancer diagnosis, due to its critical mortality rate, warrants a comprehensive, thorough review. Therefore, we will extensively analyze the current research and improvements, mainly focusing on fluorescent-based aptameric biosensors for liver cancer diagnosis. More specifically, among diverse fluorescent-based aptameric biosensors, we will discuss two detection strategies, (i) Förster resonance energy transfer and (ii) metal-enhanced fluorescence, in the following sections (Figure 1). Additionally, we will highlight the strengths and limitations of aptameric biosensors and their future challenges. We believe this review will inspire interdisciplinary attention wherein significant progress is made in liver cancer diagnosis and prognosis for successful treatment.

## 2. Förster Resonance Energy Transfer-Based Biosensors for Liver Cancer Diagnosis

### 2.1. Förster Resonance Energy Transfer

An optical process, Förster resonance energy transfer (FRET), is generated between two kinds of light-sensitive molecules, the donor fluorophore and acceptor fluorophore [23,24,25]. A fluorophore changes from a ground state to an excited state by absorption of excess energy, such as photons from their surroundings. When the fluorophore becomes an electronically excited state, the excited energy falls back to a lower state with the emission of photons. Meanwhile, if the two-fluorophore molecules are closely localized (<10 nm), one of the fluorophores with a lower excited energy state (acceptor) can accept the energy released from the donor fluorophore and emit another spectrum [26,27]. Thus, controlling the distance between two molecules is critical to obtain higher efficiency of FRET as it is inversely proportional to the distance between the donor and acceptor molecules [28,29]. As the FRET mechanism is a distance-dependent energy transfer between two closely packed fluorophores, it has been widely utilized to monitor the specific interaction between the biomolecules as well as molecular conformational changes in a precise manner.

### 2.2. FRET-Based Aptameric Biosensor for Protein Analysis

Among various biomarkers, proteins have gained extensive attention as a major biomarker to diagnose and prognosis cancers. For example, alpha-fetoprotein (AFP) is commonly used as a diagnostic marker for liver cancer patients. The presence of hepatocellular carcinoma (HCC) and chronic liver disease is often linked to elevated levels of AFP. Liver cancer patients have elevated AFP in serum up to 400 ng/mL [30] compared to healthy individuals below 25 ng/mL. Further, as the level of AFP is also related to the stage of the disease, identification of AFP is of great interest for the early diagnosis and clinical treatment of liver cancer [31].

**Figure 2:**
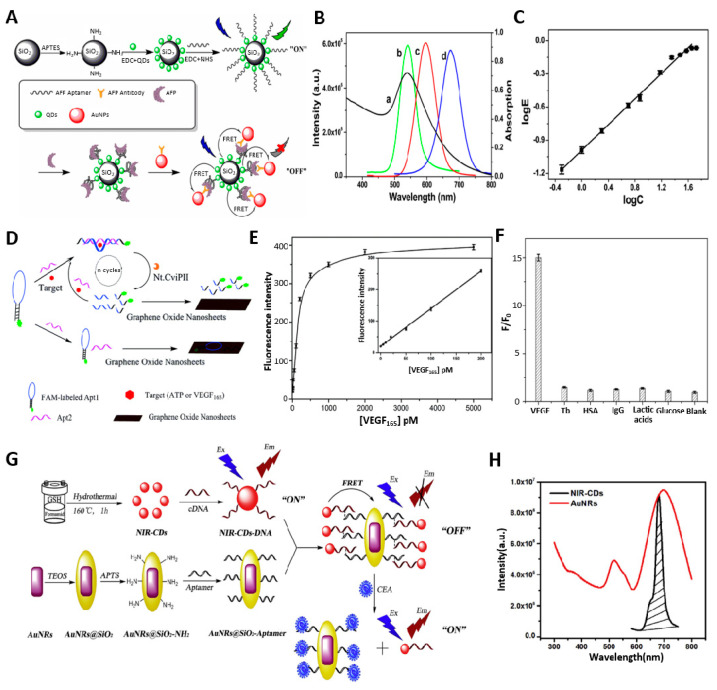
(**A**) Illustration of a fluorescent sensor based on the FRET and has a sandwich structure involving QDs, AFP, and AuNPs. (**B**) Fluorescence spectroscopy to obtain ideal donor−acceptor pairs. (**C**) The linear detection range between the energy transfer efficiency and AFP concentrations. (**D**) Schematic illustration of the process for biomolecule detection. (**E**) The fluorescence intensity with the concentration of VEGF. The inset indicates the linear relationship. (**F**) Selectivity of VEGF with other biomolecules. (**G**) NIR−CDs−based fluorescence aptasensor for CEA detection. (**H**) The overlap between the emission spectrum of NIR−CDs and the absorption spectrum of AuNRs. (**A−C**): Reproduced with permission from [32], published by Talanta 2019. (**D−F**): Reproduced with permission from [37], published by Analyst 2015. (**G**,**H**): Reproduced with permission from [38], published by Analytica Chimica Acta 2019.

By utilizing the FRET mechanism, Zhou et al. described a highly sensitive aptamer-based biosensor to detect AFP [32] (Figure 2A). In the corresponding biosensor system, luminescent CdTe quantum dots (QDs) were labeled with the AFP aptamer as a donor. Gold nanoparticles (AuNPs) were functionalized with an anti-AFP antibody as an acceptor. When the AFP is presented, the selective affinity between aptamer, AFP, and antibody causes the localization of QDs and AuNPs in proximity. This leads to quenching the fluorescent signal emitted by CdTe QDs through the FRET mechanism between the donor (QD) and acceptor (AuNP). In addition, fluorescence spectroscopy was used to examine the spectroscopic properties of QDs and AuNPs probes functionalized with antibody/aptamer to select the ideal donor–acceptor pair. As a result, apt-apt-QD1-SNP, a pair matching the absorption spectrum of the antibody functional AuNP, was selected (Figure 2B). As the described aptasensor was a fluorescence turn-off sensing system, it showed a concentration-dependent decrement of fluorescent intensity; however, it exhibited a reliable signal within the linear detection range of 0.5–45 ng/mL, and the limit of detection was found to be 400 pg/mL (Figure 2C). Conversely, Lu et al. constructed an aptamer-based sensor system with fluorescence recovering strategies by incorporating 5-carboxyfluorescein (FAM) and gold nanoclusters (AuNCs) as a donor and acceptors [33]. AuNCs can effectively quench the fluorescence of FAM while forming a complex through strong coordination interactions caused by AFP aptamers. When AFP is introduced into the system, the AFP aptamer favorably interacts with AFP. This interaction induces a conformational change of AFP aptamer, thus recovering the fluorescence of FAM by releasing AuNCs from the complex. This example has an AFP detection limit of 6.631 ng/mL, with a linear detection range from 10.0–100.0 ng/mL. Similarly, Li et al. developed an AFP biosensor using a fluorometric aptamer nanoprobe composed of 5-carboxyfluorescein and palladium nanoparticles (PdNPs) [34]. Here, the fluorescence is quenched by PdNPs adsorbed on the FAM-AFP aptamer via the nitrogen functional group. The presence of AFP promoted the conformational change of aptamer, resulting in the recovery of the fluorescence via weakened interaction between the aptamer and the PdNPs. The fluorescence recovery range related to the AFP concentration was 5.0–150 ng/mL with 1.4 ng/mL as the detection limit. The assay also showed 98.3 to 112.9% of the recovery values for spiked diluted human serum, demonstrating the possibility of the real sample analysis. Although the detection range of the above sensors does not cover up to 400 ng/mL, which is the concentration of AFP in cancer patients, it is adequate as a primary confirmation that liver problems occur in healthy people.

Vascular endothelial growth factor (VEGF) is a critical growth factor involved in regulating tumor growth and proliferation. The dysregulation of VEGF expression leads to the development of solid tumors by promoting tumor angiogenesis, as VEGF plays a crucial role in regulating angiogenesis and vascular permeability [35]. Using a FAM molecule and graphene oxide (GO), Wang et al. developed a highly sensitive biosensor that detects VEGF based on the FRET mechanism in their study [36]. For the biosensor system, anti-VEGF aptamers were labeled with fluorescent dyes and adsorbed on the GO surface by π–π interactions between the ring structure of the nucleobase of aptamer and the flat planar GO sheet. Consequently, the fluorescence of the dye was quenched by the FRET mechanism caused by fluorescent dye and GO. When it recognizes and binds to VEGF, which acts as a target, it specifically forms a VEGF–aptamer complex, releasing the VEGF–aptamer complex from the GO surface to restore the fluorescence signal of the FAM. With a detection limit of 2.5 × 10^−10^ M, the developed sensing platform demonstrated exceptional sensitivity and selectivity towards VEGF. Furthermore, the fluorescence aptasensor was tested with four human serum samples obtained from cancer patients and healthy individuals to validate its feasibility for real sample analysis. As expected, significantly higher levels of VEGF were observed in cancer patients compared to healthy individuals. Additionally, the range of 98.6–105.3% recovery of VEGF further confirmed the accuracy of the aptasensor for detecting VEGF in clinical samples. The Fan research group also developed an amplified fluorescence aptasensor platform for detecting VEGF and ATP [37] (Figure 2D). In this system, a FAM labeled molecular aptamer beacon was utilized as the recognition element, and GO was employed as a fluorescence quencher, while signal amplification was obtained by catalytic reaction through nicking endonuclease enzyme. When the target makes a complex with an aptamer via a duplex DNA region, the nicking enzyme can recognize and cleave the specifically designed site of the aptamer. The cleavage of the aptamer could lead to the formation of one short (8 bases) DNA fragment with a FAM label and one long (22 bases) DNA fragment. Consequently, a shortage of DNA base pairs will reduce binding affinity to the GO, thus gradually increasing fluorescence intensity. Under optimized conditions, the proposed platform exhibited promising results for quantifying VEGF with a detection limit as low as 1 pM (Figure 2E). In addition, as a result of simultaneous exposure with other biomolecules, the VEGF fluorescence intensity was maintained high even though the concentration was lower than that of other tested analytes. The results showed that the assay for VEGF was highly selective (Figure 2F).

Carcinoembryonic antigen (CEA) is also one of the most widely used tumor markers for diagnosis and prognosis in cancer patients [38,39]. Though the carcinoembryonic antigen (CEA) is mostly expressed in colorectal cancers (CRCs), including gastrointestinal, breast, and lung cancers, the elevation in the level of CEA is also a significant analytical indicator for liver metastasis, which is the leading cause of death from colorectal cancer [40]. The normal level of CEA in healthy adults is less than 5 ng/mL, but in a patient with metastatic liver cancer, there is a significant rise in the concentration of CEA [41]. Shao et al. constructed a highly sensitive aptasensor for detecting CEA by utilizing the FRET mechanism between near-infrared carbon dots (NIR-CDs) and gold nanorods (AuNRs) [38] (Figure 2G). The high signal-noise ratio (SNR) could be obtained as the aptasensor was fabricated by NIR-CDs to avoid the inherent auto-fluorescence of biological samples effectively. For this purpose, a one-step solvothermal method was employed to synthesize the highly water-dispersible NIR-CDs (λmax ¼ 680 nm). Tuning the aspect ratio of AuNRs allowed their absorption bands to overlap with the emission spectra of NIR-CDs, enabling efficient FRET (Figure 2H). With the help of aptamer, the CEA was successfully detected with a linear range of 0.1–5 ng/mL and a detection limit of 0.02 pg/mL. In another example, Zhou and his coworkers developed a FRET-based aptameric sensor to detect Glypican-3 (GPC3) [42]. GPC3, one of the oncofetal transmembrane proteoglycans, has also been confirmed to be closely related to cancer cells’ growth, proliferation, invasion, and metastasis [43]. Notably, GPC3 is overexpressed in hepatocellular carcinoma (HCC) and is a biomarker for HCC diagnosis [43]. The aptasensor was designated with GPC3 aptamer labeled gold carbon dots (Au-CDs) and magnetic Fe_3_O_4_ nanoparticle-decorated graphene oxide (Fe_3_O_4_/GO) nanosheets. Once the GP3 aptamer is released from the Fe_3_O_4_/GO nanosheets through the conformational change that occurred by specific binding with GP3, the leftover quencher, Fe_3_O_4_/GO nanosheets, can be magnetically removed. As a result, fluorescence from the free AuCDs-GPC3 Aptamer complex can be solely restored without interferences from residual quenchers. The fluorescence recovery was linearly correlated in the 5–100 ng/mL range, and the detection limit could reach 3.01 ng/mL.

### 2.3. FRET-Based Aptameric Biosensor for miRNA Analysis

Recently, microRNA (miRNA), a type of small regulatory RNA, has gained significant attention as one of the potential biomarkers for cancers due to their important functions in development and diseases [44]. Notably, the dysregulated miRNAs have been shown to affect the proliferative signal to induce undesired angiogenesis and lead to metastasis. To this end, compelling evidence has identified miRNAs as potential biomarkers to investigate for human cancer diagnosis and prognosis. For example, numerous studies have shown that miR-10b, miR-21, miR-222, and miR-224 are up-regulated, while miR-122 and miR-200 are down-regulated in HCC patients [45,46].

**Figure 3:**
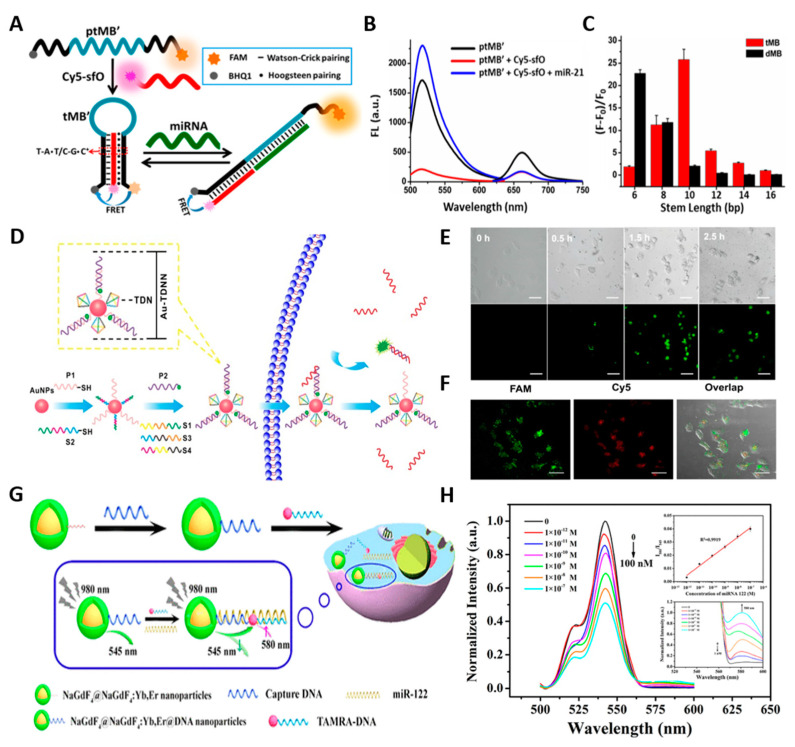
(**A**) Schematic illustration of the detection of miRNA using the tMB. (**B**) Fluorescence emission spectra of tMB formation and target addition. (**C**) The fluorescence recovery degrees according to different triplex or duplex lengths. (**D**) Structure and working principle of Au−TDNNs. (E) Uptake efficiency and dynamics of Au−TDNNs in the presence of miR−21 in live cells. Scale bars = 50 µm. (**F**) Strong FRET signal (red fluorescence) for miR−21 in HepG2 cells. Scale bars = 50 µm. (**G**) Illustration of the Synthesis and Workflow of UCNPs@DNA Nanoparticles for Sensitive Detection of miR−122. (**H**) Emission spectra of UCNPs@DNA nanoparticles with different concentrations of miR−122. The insets show the linear relationship (up) and the enlarged view of the emission at 580 nm (down). (**A**–**C**): Reproduced with permission from [47], published by ACS Sensors 2018. (**D**–**F**): Reproduced with permission from [48], published by Theranostics 2018. (**G**,**H**): Reproduced with permission from [51], published by ACS Applied Materials & Interfaces 2018.

Lu et al. designed triplex molecular beacons (tMBs) to construct the FRET-based biosensor for miRNA-21 [47] (Figure 3A). Molecular aptamer beacons typically comprise a stem, loop, fluorophore (donor), and quencher (acceptor). Upon binding with targets, the fluorophore and quencher of MBs are separated via a loop opening, therefore recovering the fluorescence signal (Figure 3B). Generally, the stem structure of MBs usually consists of two complementary sequences bound by hydrogen bonds. Even though the strength and stability of the stem structure can be achieved by controlling the length of base pairs (bps), it is also considered a limitation to meet the requirements for various targets, as the best stability can be attained with 5–7 bps. Therefore, to target the complementary sequence of miR-21 with the loop structure, tMBs were carefully designed with a triplex stem structure containing protonated cytosine–guanine–cytosine (C-G•C+) and thymine-adenine-thymine (T-A•T). Comparing the fluorescence intensities with dMBs according to the duplex length demonstrated that tMB has better controllability by nucleotide sequence (Figure 3C). Owing to the design of tMBs, the binding strength of the stem structure was controlled by utilizing both Watson–Crick and Hoogsteen base-pairings. When miR-21 is added, only the Hoogsteen pairing is broken to create a rigid heterozygous hybrid duplex structure, hindering FRET between the fluorophore (FAM) and quencher (BHQ1), resulting in fluorescence recovery. The use of fluorescence recovery allows for measuring the miR-21 concentration in the linear range from 0.5 to 250 nM, with a detection limit of 0.18 nM. Furthermore, by introducing simple modifications to the tMB sequence, the proposed tMBs could be used in the stem design of MBs to detect protein biomarkers such as VEGF, indicating their potential feasibility for various applications.

Bai et al. utilized a tetrahedral DNA nanostructure (TDN) combined with gold nanoparticles (AuNPs) to fabricate a FRET-based nanosensor (Au-TDNN) for intracellular miRNA detection [48] (Figure 3D). Fluorescent dye-labeled double-stranded probes were thermodynamically designed to achieve specific measurements of miR-21. In addition, AuNPs were specifically utilized to provide not only the fluorescence quenching effects, but also a large surface area for TDN adhesion (binding ratio 1:27). Furthermore, excellent cellular uptake efficiency could be achieved through the TDN structure [49] and the phosphorothioate-modification enhanced stability against enzymatic degradation, significantly reducing false-positive signals [50]. Consequently, this allows the detection of miR-21 in both MCF-7 cell extracts and hepatocyte cancer cells (HepG2) (Figure 3E, F). In another approach, Ren et al. created a simple FRET-based biosensor to detect liver-specific miR-122 using DNA-functionalized core/shell structured upconversion nanoparticles (UCNPs) (NaGdF4@NaGdF4:Yb, Er) as energy donors [51] (Figure 3G). A notable aspect of the study is that the DNA aptamer was attached directly to the hydrophobic UCNPs using a one-pot ligand exchange process at the interface between two liquids. Using a sandwich approach involving capture DNA, dye-labeled report DNA, and target miR-122, control of the distance between the donor and acceptor was possible, resulting in improved FRET efficiency and specificity. Based on this approach, researchers have detected miR-122 at a limit of detection of 10^−13^ M, with a linear detection range from 0–10^−12^ M (Figure 3H). This technique has also been further utilized to perform imaging of HepG2 cancer cells and in vivo imaging of a nude mouse that had been inoculated with HepG2 cells. The recent research on FRET-based aptameric biosensors for cancer diagnosis is compared in Table 1.

## 3. Metal-Enhanced Fluorescent-Based Analysis Methods for Liver Cancer Diagnosis

### 3.1. Metal-Enhanced Fluorescent

MEF is a technique that enhances fluorescent intensity by positioning metallic nanostructures near fluorophores, thereby enabling the detection of ultra-low biomarker concentrations through surface plasmon resonance (SPR) [17]. SPR refers to the excited state of surface plasmons when light is incident on a flat surface of noble metals such as gold (Au), silver (Ag), platinum (Pt), etc. [55]. Strong and confined electromagnetic fields are induced by the incidence of light on the nanostructure formed by these noble metals due to localized surface plasmon resonance (LSPR). These electromagnetic fields facilitate additional electronic configurations in nearby fluorophores, thereby increasing the rate of excitation and emission. The physical properties of the metal nanostructure have been considered as among the critical factors affecting the enhancement of the fluorophore. In addition, the coupling interaction between the noble metal nanostructure and fluorophores also influences the MEF effect. The distance (5–90 nm) and the absorption/emission spectral overlap between the metal NPs and fluorophores are key factors to take full advantage of the MEF effect. Although the maximum enhancement is known to occur at approximately 10 nm distance between the metal nanostructure and fluorophore, fluorescence can be quenched when the fluorophore is excessively close to the metal nanostructure [18]. Owing to the unique phenomena of MEF, various approaches have been developed to amplify biosensor signals more effectively and characterize biomarkers more precisely.

### 3.2. MEF-Based Aptameric Biosensor for Protein Analysis

**Figure 4:**
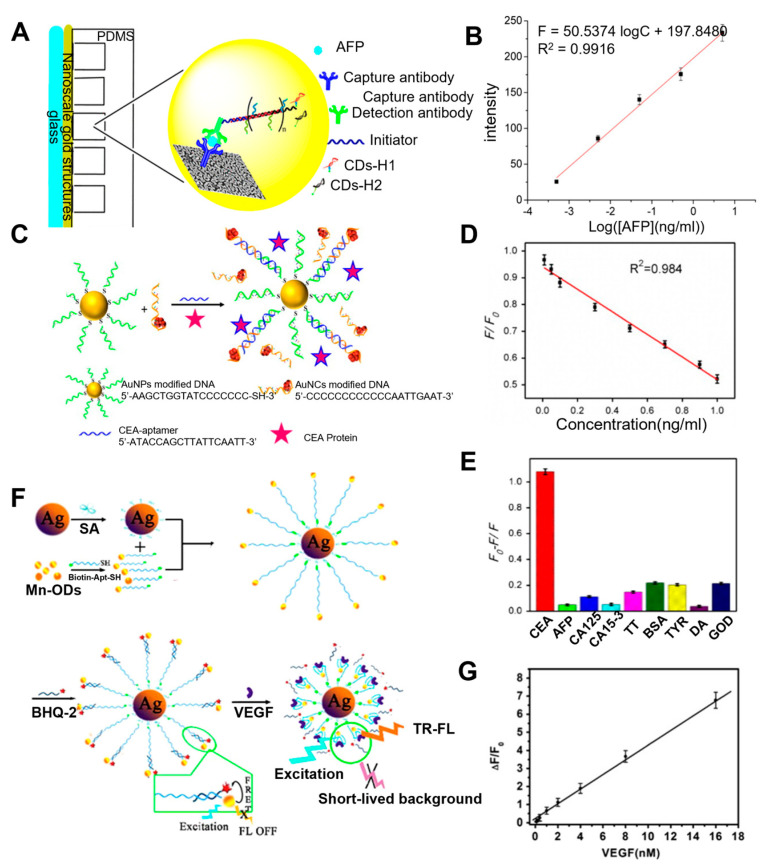
(**A**) Schematic illustration of the operation to detect AFP with the Immuno−HCR and MEF of Carbon Dots. (**B**) Linear plot for fluorescence corresponding to concentrations of AFP. (**C**) Schematic illustration of the MEF strategy based on the two different types of nanomaterials for assaying CEA. (**D**) Linear plot of F/F_0_ versus concentrations of CEA from 0.01 ng/mL to 1 ng/mL. (**E**) Selectivity of CEA (0.8 ng/mL) with other related proteins. (**F**) Schematic illustration of the fabrication procedures of the silver nanoparticle−enhanced TR−FL sensor based on the Mn−doped ZnS QDs and the detection mechanism for VEGF_165_. (**G**) Linear plot of ΔF/F0 to VEGF_165_ concentration and TR-FL sensor specificity for VEFG_165_ (**A**,**B**): Reproduced with permission from [56], published by ACS Appl. Mater. Interfaces 2017. (**C**–**E**): Reproduced with permission from [58], published by ScienceDirect Biosensors and Bioelectronics 2015. (**F**,**G**): Reproduced with permission from [59], published by ScienceDirect Biosensors and Bioelectronics 2015.

A few studies have reported on developing aptamer-based analysis methods based on the MEF mechanism for liver cancer diagnosis. Tang and his group have developed a dual amplification fluorescence sensor by combining immuno-hybridization chain reaction (Immuno-HCR) and MEF effect to detect AFP [56]. The sandwich structure was formed by the immobilized antibody on the plasmonic slide, target molecule (AFP), and detection antibodies conjugated with an oligonucleotide initiator. Then, carbon dot (CD)-tagged DNA hairpins (H1 and H2) complementary to the oligonucleotide initiator were introduced to prompt the HCR (Figure 4A). As a result, a multiple repeat unit of a copolymer was generated to amplify the fluorescent signal from CDs. CDs are one kind of fluorescent nanomaterials that have received much attention due to their small size, light bleaching prevention, and biocompatibility [57]. Through these dual amplifications, exceptional sensitivity for AFP was obtained at a detection limit of 94.3 fg/mL with wide linearity (0.0005–5 ng/mL) (Figure 4B).

Furthermore, photostable fluorescent materials are desired to avoid photolysis associated with conventional fluorophores. To this end, Yang et al. demonstrated the use of silver nanoclusters (AgNCs) as fluorescence probes to analyze carcinoembryonic antigen (CEA) selectively [58]. The binding aptamer for CEA was critically designed with 25 bases to connect the fluorophore (AgNCs) and enhancer (AuNps) with an estimated distance of around 10 nm to provide enhanced fluorescence appearing via the MEF effect. However, adding CEA promoted the competitive binding with the AuNPs modified detection aptamer, half-complementary to the CEA binding aptamer, which disturbed the MEF effect (Figure 4C). Thus, the presence of CEA could be quantified as it was proportional to the decrease in fluorescence. The detection limit of this sensor was 3 pg/mL at a signal-to-noise ratio of 3 (Figure 4D). The developed biosensor also demonstrated highly selective detection of CEA from the mixture with highly concentrated other biologically relevant proteins such as AFP, CA125, CA15-3, thrombin, Bovine serum albumin (BSA), tyrosinase, dopamine, glucose oxidase. Furthermore, recoveries (98.16%, 102.5%, 95.31%) from human serum also exhibited insignificant interference from substrates of the blood as well (Figure 4E).

In another approach, Zhu et al. utilized long-lived fluorescence QDs (Mn-doped ZnS) with AgNps to develop a more sensitive time-resolved fluorescence (TR-FL) biosensor for the detection of VEGF_165_ [59]. The QD-modified aptamers were bonded to AgNPs through streptavidin–biotin interaction, while BHQ-2 quencher-labeled DNA strands hybridized with aptamer to form a duplex structure. The weak fluorescence intensity was observed in the duplex structure due to the FRET between localized QDs and BHQ-2 quenchers. In the presence of VEGF_165_, the displacement of the quencher-labeled strands disrupted FRET between QDs and BHQ-2 quenchers, thereby recovering fluorescence. The binding of VEGF_165_ to aptamer not only released the quencher from the duplex structure but also promoted the structural changes of the aptamer, which led towards the close localization of QD near the AgNPs. Therefore, the fluorescence of the QDs could amplify the MEF effect within the proximately localized AgNPs (Figure 4F). Unlike the conventional fluorescence sensor, the AgNPs-enhanced TR-FL sensor shows advantages in terms of sensitivity and high signal-to-noise ratio as the fluorescence signal can be measured without the interference of short-lived background by adjusting delay and gate time. To this end, VEGF_165_ could be analyzed in a linear range of 0.1 nM to 16 nM with 0.08 nM as the detection limit (Figure 4G).

### 3.3. MEF-Based Aptameric Biosensor for miRNA Analysis

Following the initial development of an RNA sensor via the MEF effect [60], tremendous approaches have been made to analyze specific RNAs [61,62]. However, despite these advances, limited studies have reported aptamer-based miRNA analysis methods specifically for liver cancer diagnosis. This highlights the need for further research and development in this area.

**Figure 5:**
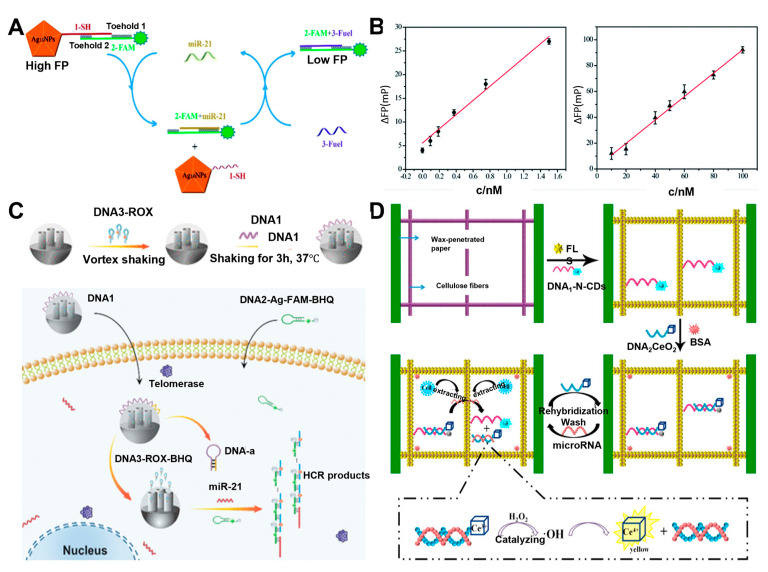
(**A**) Schematic illustration of biosensor based on Cyclic strand displacement reaction for detecting miR−21. (**B**) Linear plot of the fluorescence polarization change (ΔFP) corresponding to the concentrations of miRNA−21 with and without cyclic strand displacement reaction. (**C**) Schematic of the structure and operation of FOMN−based dual−signal logic. (**D**) Schematic illustration of the fabrication procedures and detection mechanism. (**A**,**B**): Reproduced with permission from [63], published by RSC Advances 2020. (**C**): Reproduced with permission from [64], published by ACS Appl. Mater. Interfaces 2021. (**D**): Reproduced with permission from [67], published by ScienceDirect Biosensors and Bioelectronics 2017.

Zhu and his group developed the enzyme-free fluorescence biosensor for detecting miR-21 via the MEF mechanism [63]. A biosensor probe was developed by combining Ag_10_NPs and a nucleic acid strand (named 1-SH) that can form a complementary structure with a fluorescent dye-labeled strand (named 2-FAM). In the presence of target miRNA-21, the strand displacement reaction occurs, exposing the toehold of 2-FAM to react with the nucleic acid strand (named 3-fuel). Consequently, the miRNA-21 could be released to promote the next cycle of displacement reaction of 2-FAM from the probe again (Figure 5A). As a result, a certain distance between the fluorophore (FAM) and Ag_10_NPs cannot be maintained, thus leading to a decrement in the fluorescence signal (Figure 5B). Using the fluorescence difference between the initial and final product, the presence and concentration of miR21 could be successfully characterized from the buffer and real samples in the linear range of 100 pM to 16 nM with a detection limit of 93.8 pM.

In addition, incorporating suggested biosensing strategies on the microfluidic chip improved sensitivity by reducing the reagent amount and the reaction time and simplified the overall process. In their work, Shi et al. also assembled a dual-signal (fluorescence and Raman signals) analysis by designing a signal amplification strategy via functionalized ordered mesoporous nanoparticles (FOMNs) based on Boolean logic “AND” [64]. The dual signals could only be observed under the presence of both telomerase and miR-21. Briefly, the presence of telomerase worked as an input to release the DNA-ROX (carboxy-X-rhodamine)-BHQ hairpin complex from sFOMNs. After DNA-Ag and DNA-ROX-BHQ hybridized in the presence of miR-21 as the second input, the HCR process subsequently trailed to amplify both fluorescence and Raman signals. Additionally, both fluorescence and Raman signals were enhanced by the LSPR mechanism obtained by AgNPs (Figure 5C). Experimental output reflected dual signals obtained from suggested strategies were relevant to the expression levels of telomerase and miR-21 in living cells.

In another approach, Cui’s group employed AgNPs on a microfluidic chip to detect miR-21 through MEF and surface-enhanced Raman scattering (SERS) spectroscopies [65]. The chip was fabricated by immobilizing AgNps and followed by specifically designed MBs. A thiol decorated on the 3′ ends of the MB facilitates the binding of MB on AgNPs, while an organic dye (6-FAM) on the 5′ ends of the MB work as both a fluorescence label and SERS reporter. The 6-FAM labeled on the MB localized to the nearby AgNPs due to the simultaneous formation of the hairpin structure. The raman signal was enhanced without the presence of the target molecule, while the fluorescence signal was quenched. Oppositely, with the presence of target miR-21, the hairpin structure is unfolded by the hybridization, resulting in a recovery of fluorescence and decrement of SERS signal due to the increased distance between 6-FAM and the AgNPs. An improved sensitivity and linear feature (0–10^−7^ M) were obtained by combining the opposite changing trends induced by the target. Similarly, Masterson et al. also developed a biosensor for detecting microRNA using chemically synthesized gold triangular nanoprisms (Au TNPs), which can be used for LSPR-based SERS and MEF mechanisms [66]. Liang et al. proposed an enhanced fluorescence/visual bimodal biosensor for multiplexed detection of miRNA (miR210 and miR21) by incorporating a flower-like silver (FLS) structure fabricated microfluidic paper-based analytical devices (µPADs) [67]. Nitrogen-doped carbon nanodot (N-CDs) functionalized DNA strand (DNA1-N-CDs) was adhered on the FLS and hybridized with a quencher (CeO_2_) modified DNA strand (DNA2-CeO_2_) to fabricate enhanced fluorescence biosensor. Upon the addition of the target miRNA, DNA2-CeO_2_ was released, and nearby FLS could dramatically strengthen the fluorescent intensity of leftover DNA1-N-CDs. On the other hand, detached DNA2-CeO_2_ also served as an enzyme based on their oxidase activity, resulting in a color change for real-time visual analysis of miRNA (Figure 5D). Due to the background fluorescence and scattering from the additives and paper substrate, it is hard to recognize the fluorescence signal directly from μPAD. However, the implication of FLS on µPADs reduced the background fluorescence and provided surface enhanced fluorescence for sensitive and accurate determination of miRNAs. Additionally, the multi-channel µPADs design allowed the multiplexed detection of miRNA, and the µPADs could be recycled by replacing supplementation of DNA2-CeO_2_ and visual substitutive. By combining these strategies, enhanced fluorescence and bimodal visual platform, miRNA-21 and miRNA-210 were detected as low as 0.06 fM and 0.03 fM, respectively. Although there are many studies on MEF-based biosensors, we present a summary of exciting approaches to analyze specific biomarkers (protein and miRNA) related to liver cancers (Table 2).

## 4. Conclusions

The difficulty of early detection contributes to the high mortality rate of liver cancer, presenting a formidable challenge to medical professionals worldwide. This review has shed light on the recent advancements in the robust FRET and MEF biosensing systems, which leverage aptamer-based nanotechnology to detect biomarkers associated with liver cancer. These fluorescent-based approaches have shown promising results in the sensitive and selective detection of various liver-cancer-related biomarkers, including proteins and miRNA. However, while the proof-of-concept strategies for developing aptamer-based biosensors are undoubtedly exciting, the journey towards their real-world clinical application is still ongoing. The key to this journey is the optimization of the aptamer selection process. The choice of aptamers is critical, as it affects two crucial factors: control of the distance between molecules and selective binding to the target. Advancements in the development of aptamers, which could allow for the precise detection of various cancer biomarkers, could revolutionize cancer diagnosis by improving the accuracy and precision of cancer diagnostics through multiplexing to provide a more comprehensive understanding of the disease. However, these promising biosensors have yet to see widespread adoption in clinical settings. Therefore, comprehensive and systematic clinical trials are essential to validate their efficacy and safety in real-world situations.

While the primary focus of this review was on FRET and MEF, other optical technologies such as resonance and colorimetric methodologies also have a role to play in diagnosing prevalent cancers such as breast, lung, and prostate cancer. Resonance-based methods, including surface plasmon resonance (SPR) and localized surface plasmon resonance (LSPR), are appreciated for their rapid, real-time analysis capabilities. However, they also face limitations such as low specificity and the need for complex instrumentation. On the other hand, surface-enhanced Raman spectroscopy (SERS) exhibits superior specificity and excellent photophysical properties, though it requires bulky optical components. Lastly, colorimetric techniques are simple and do not require costly separate analysis devices but suffer from low sensitivity. Each of these optical technologies, with their unique strengths and weaknesses, indicates that the field of optical biosensing is a diverse and evolving landscape. In conclusion, continued refinement and enhancement are needed to fully exploit their potential in the fight against cancer.

## Figures and Tables

**Table 1:** FRET-based aptameric biosensors for cancer diagnosis.

Detection Strategy	Target Biomarker	Linear Range	LOD	Reference
CdTe quantum dots (QDs) labeled AFP aptamer as a donor and gold nanoparticles (AuNPs) functionalized anti-AFP antibody as an acceptor.	AFP	0.5–45 ng/mL	400 pg/mL	[32]
Incorporating 5-carboxyfluorescein (FAM)–APF aptamer and gold nanoclusters (AuNCs) as a donor and acceptors	AFP	10.0–100.0 ng/mL	6.631 ng/mL	[33]
FAM-AFP aptamer as donor and PdNPs as acceptor)	AFP	5.0–150.0 ng/mL	1.38 ng/mL	[34]
Fluorescent dye-labeled anti-VEGF aptamer as a donor and GO as an acceptor	VEGF	5 × 10^−10^ −5 × 10^−9^ M	2.5 × 10^−10^ M	[36]
FAM-labeled Apt1 as a donor and GO as a super-quencher	VEGF	5–200 pM	1 pM	[37]
Energy donor, Fluorophore (FAM), and quencher (BHQ1)	VEGF	0.05–6 ng/mL	-	[47]
Near-infrared carbon dots (NIR-CDs) as donors and gold nanorods (AuNRs) as acceptors	CEA	0.1–5000 pg/mL	0.02 pg/mL	[38]
GPC3 aptamer labelled gold carbon dots (AuCDs-GPC3_Apt_) as a donor and magnetic graphene oxide (Fe_3_O_4_/GO) nanosheets as an acceptor	GPC3	5–100 ng/mL	3.01 ng/mL	[42]
Energy donor, fluorophore (FAM), and quencher (BHQ1)	miR-21	0.5–250 nM	0.18 nM	[47]
Fluorescent dye-labeled detection probe on Au-TDNNs as donor and Au-NPs as an acceptor	miR-21	-	-	[48]
DNA-functionalized UCNPs were designed as energy donors, and TAMRA labeled on another shorter DNA as the energy acceptor	miR-122	0–10^−12^ M	10^−13^ M	[51]
FAM-labeled probe as the donor and MoS_2_–PEG–FA nanosheets as an acceptor	miR-21	-	-	[52]
Dual-signal-tagged chimeric DNA probe (dcDNA) as donor and PAA-Ti_3_C_2_ as an acceptor	miR-21	0–25 nM	0.8 nM	[53]
BHQ-3-induced quenching of AP-DNA-fluorescent Cy5	miR-21			[54]

**Table 2:** MEF-based aptameric biosensors for cancer diagnosis.

Detection Strategy	Target Biomarker	Linear Range	LOD	Reference
Dual amplification by immunohybridization chain reaction(immune-HCR) and metal-enhanced fluorescence with carbon nanodots (CDs)	AFP	0.0005–5 ng/mL	94.3 fg/mL	[56]
Surface-enhanced fluorescence (SEF) strategy based on the two types of nanomaterials, gold nanoparticles and silver nanoclusters	CEA	0.01–1 ng/mL	3 pg/mL	[58]
Mn-doped ZnS quantum dots labeled AgNPs enhanced time-resolved fluorescence sensor for improving sensitivity to detect ${\mathrm{VEGF}}_{165}$	VEGF	0.1–16 nM	0.08 nM	[59]
Biosensor combined with reconstructive molecular beacon for detecting miRNA	miR-21	10 fM–100 pM	1.38 fM	[61]
Cyclic strand displacement reaction with AgNPS and tree nucleic strand(2-FAM, 3-fuel, 1-SH) for detecting miR-21 with high effectiveness	miR-21	0.16–16 nM	93.8 pM	[63]
FOMN-based dual-signal logic operation strategy for detection of cancer biomarker microRNA	miR-21	2 pM–1 nM	0.05 fM	[64]
The combination of fluorescence and surface-enhanced Raman scattering techniques for improving the sensitivity of detection of micro-RNA	miR-21	0–10^−7^ M	-	[65]
Chemically synthesized gold triangular nanoprisms (Au TNPs) for LSPR-based SERS and PEF mechanism to detect microRNA	miR-10b, miR-96	-	1.13 pM, 0.030 pM	[66]
Flower-like silver (FLS)-enhanced fluorescence/visual bimodal platform for multiple miRNAs	miR-21	0.2 fM–2 nM	0.06 fM	[67]
Nanogap antennas with strand displacement for detecting low concentrations of nucleic acid biomarkers	miR-21	-	0.0972 fM	[68]

## Data Availability

Not applicable.
